# A novel population of memory‐activated natural killer cells associated with low parasitaemia in *Plasmodium falciparum*‐exposed sickle‐cell trait children

**DOI:** 10.1002/cti2.1125

**Published:** 2020-04-02

**Authors:** Claire Loiseau, Ogobara K Doumbo, Boubacar Traore, Jamie L Brady, Carla Proietti, Karina P de Sousa, Peter D Crompton, Denise L Doolan

**Affiliations:** ^1^ Centre for Molecular Therapeutics Australian Institute of Tropical Health & Medicine James Cook University Cairns QLD Australia; ^2^ Mali International Center of Excellence in Research University of Sciences, Technique and Technology of Bamako Bamako Mali; ^3^ Malaria Infection Biology and Immunity Section Laboratory of Immunogenetics National Institute of Allergy and Infectious Diseases National Institutes of Health Rockville MD USA; ^4^ Present address: School of Life and Medical Sciences Biosciences Research Group University of Hertfordshire Hatfield UK

**Keywords:** haemoglobin AS, natural killer cells, parasite burden, *Plasmodium falciparum*, protective immunity, sickle‐cell trait phenotype

## Abstract

**Objectives:**

The sickle‐cell trait phenotype is associated with protection from malaria. Multiple molecular mechanisms have been proposed to explain this protection, but the role of the host immune system has been poorly investigated. We hypothesised that cellular immunity to malaria may develop differently in sickle‐cell trait children (HbAS) and children with normal haemoglobin (HbAA) repeatedly exposed to *Plasmodium falciparum* (*Pf*).

**Methods:**

Paired samples collected prior to the *Pf* transmission season and during the first malaria episode of the ensuing transmission season from HbAS and HbAA children were analysed by multiplex bead‐based assay and comprehensive multi‐dimensional flow cytometry profiling.

**Results:**

Cellular immune profiles were enriched in HbAS relative to HbAA children before the start of the *Pf* transmission season, with a distinct NK subset. These cells were identified as a novel subset of memory‐activated NK cells characterised by reduced expression of the ecto‐enzyme CD38 as well as co‐expression of high levels of HLA‐DR and CD45RO. The frequency of this NK subset before the transmission season was negatively correlated with parasite density quantified during the first malaria episode of the ensuing transmission season. Functional assessment revealed that these CD38^dim^ CD45RO^+^ HLA‐DR^+^ NK cells represent a important source of IFN‐γ.

**Conclusion:**

Our data suggest that this novel memory‐activated NK cell subset may contribute to an accelerated and enhanced IFN‐γ‐mediated immune response and to control of parasite density in individuals with the sickle‐cell trait. This distinct cellular immune profile may contribute to predispose HbAS children to a relative protection from malaria.

## Graphical abstract

We present the first evidence for host cellular immunity in HbAS‐mediated relative protection from malaria. We identify the key cell population as a novel population of memory‐activated natural killer cells, which are a major source of IFN‐γ and associate with low parasitaemia in sickle‐cell trait children repeatedly exposed to *Plasmodium falciparum*. Our data suggest that these cells present before the start of the transmission season may contribute to an accelerated and enhanced IFN‐γ‐mediated immune response and to control of parasite density during the first malaria episode of the ensuing season.

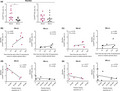

## Introduction

The sickle‐cell trait phenotype results from the heterodimerisation of normal haemoglobin (Hb) A with Hb presenting the β6 Glu → Val single amino acid substitution known as HbS. Whereas the homodimerisation of mutated HbS is responsible for the sickle‐cell disease, which is characterised by a chronic haemolytic anaemia, no severe conditions are associated with the sickle‐cell trait phenotype (HbAS). On the contrary, it is well established that individuals carrying the heterozygous form HbAS are relatively protected from malaria.^1^, ^2^ Specifically, HbAS individuals display a reduced mortality to *Plasmodium falciparum* (*Pf*) infection,^3^ a reduced parasitaemia during symptomatic malaria^1^, ^4^, ^5^, ^6^, ^7^, ^8^ and a delayed onset of malaria,^8^, ^9^ as compared with HbAA individuals. Moreover, the frequency of HbAS individuals is particularly high in areas with endemic *Pf* transmission.^5^, ^10^

A number of molecular mechanisms have been proposed to explain the relative protection from malaria displayed by HbAS individuals, including parasite growth inhibition in hypoxic condition,^11^, ^12^, ^13^, ^14^ enhanced splenic clearance,^13^, ^15^, ^16^ altered cytoadherence,^17^, ^18^, ^19^ translocation of HbS‐specific parasite growth‐inhibiting microRNAs,^20^ induction of haem oxygenase‐1^21^ and HbS polymerisation‐dependent parasite growth inhibition.^18^ A role for the host immune system in the sickle‐cell trait phenotype‐associated protection has been tentatively proposed, but this has been poorly explored.^22^, ^23^, ^24^, ^25^ Few studies have considered the importance of humoral immunity,^26^, ^27^, ^28^, ^29^, ^30^ but to the best of our knowledge, there has been no investigation of the role of host cellular immunity in the relative protection of HbAS individuals from malaria.

In malaria‐endemic area, naturally acquired immunity is known to develop slowly with age and exposure.^31^, ^32^, ^33^ This immunity includes anti‐disease or clinical immunity, which protects against disease symptoms, and anti‐parasite immunity, which limits blood‐stage *Plasmodium* burden. IFN‐γ, a key cytokine of the systemic immune response produced by both innate and adaptive immune cells, has been implicated in the protective immunity to *Pf* infection.^34^, ^35^, ^36^, ^37^, ^38^, ^39^ In humans, IFN‐γ concentration is correlated with protection from symptomatic malaria^40^ and resistance to *Pf* reinfection.^41^, ^42^ *Ex vivo* studies have demonstrated that natural killer (NK) cells are the major source of IFN‐γ during the very early immune response following *Pf* infection.^43^, ^44^ Although NK cells have been traditionally defined as innate lymphocytes, the existence of memory‐like NK subsets, which display an accelerated and enhanced recall response following re‐stimulation, has been recently recognised.^45^, ^46^, ^47^ In the context of *Pf* infection, a memory‐like status of NK has been suggested based on their adaptive T‐cell‐dependent IFN‐γ response^38^ and their antibody‐dependent cellular toxicity towards parasitised red blood cells.^48^ Most recently, in a Malian cohort of *Pf*‐exposed individuals, the frequency of an adaptive NK subset has been correlated with protection from malaria.^49^ Nonetheless, the IFN‐γ production and frequency of IFN‐γ‐producing cells, especially of NK cell subsets, have yet to be explored in the context of malaria immunity in HbAS individuals.

In this study, we hypothesised that cellular immunity to malaria may develop differently in HbAS and HbAA children repeatedly exposed to *Pf*. Accordingly, we compared IFN‐γ‐associated immune profiles of HbAS and HbAA children from a malaria‐endemic area of Mali by evaluating (1) the capacity of peripheral blood mononuclear cells (PBMCs) to respond to stimulation, and (2) the frequency of IFN‐γ‐producing cell subsets in total PBMCs, using paired samples collected prior to the *Pf* transmission season as well as during the first malaria episode of the ensuing transmission season. We then focused our work on NK cells, which were preferentially associated with sickle‐cell trait‐mediated protection, and identified a distinct subset of NK cells that was specifically enriched in HbAS children compared to HbAA children before the start of the *Pf* transmission season. We defined the phenotypic profile of these NK cells as CD38^dim^ CD45RO^+^ HLA‐DR^+^ and characterised their functional profile.

## Results

### HbAS children displayed an alteration of their systemic inflammatory response before the start of the *Pf* transmission season

**Figure 1:**
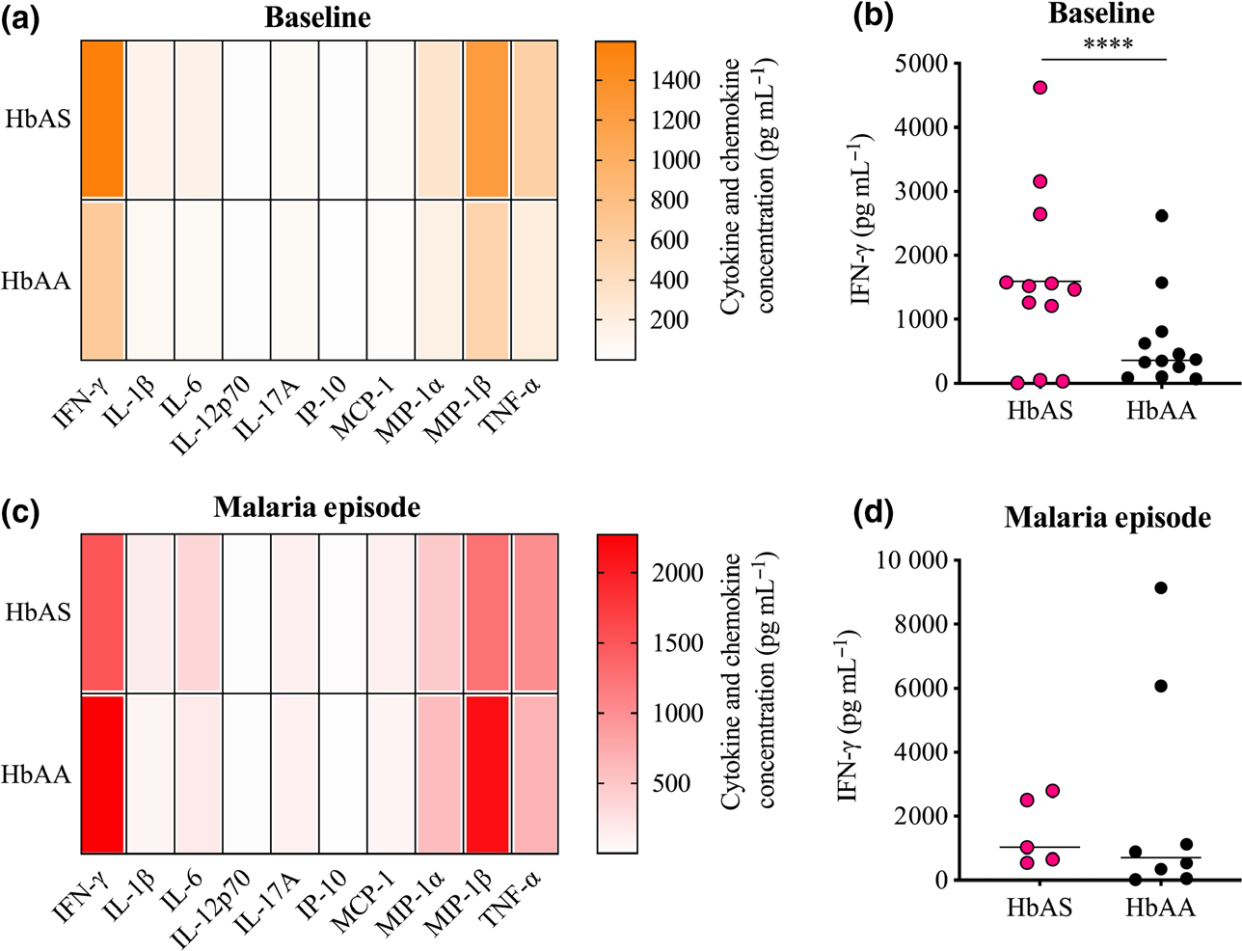
Cytokine and chemokine production in HbAS and HbAA children at baseline and during the first malaria episode of the ensuing *Pf* transmission season. **(a–d)** Concentrations of cytokines and chemokines quantified in the supernatant of stimulated PBMCs isolated from **(a, b)** HbAS children (*n* = 12) and HbAA children (*n* = 12) at baseline; or from **(c, d)** HbAS children (*n* = 5) and HbAA children (*n* = 8) during the first malaria episode of the ensuing *Pf* transmission season. Cytokine and chemokine concentrations were assessed by MagPIX multiplex bead assays. Groups were compared by Bonferroni’s multiple comparison test. ****P < 0.0001. **(a, c)** Heat maps represent the group means. **(b, d)** Horizontal bars represent the median of the group. Technical replicate (*n* = 1).

To investigate potential immune mechanisms involved in the relative protection from malaria associated with the sickle‐cell trait phenotype, we first compared the capacity of PBMCs isolated from HbAS and HbAA children repeatedly exposed to *Pf* to respond to stimulation. PBMCs collected before the start of the *Pf* transmission season (baseline) and during the first malaria episode of the ensuing transmission season (malaria episode) were stimulated *in vitro*, and the concentration of a broad range of cytokines and chemokines was measured in supernatants. Of all the cytokines and chemokines tested, only IFN‐γ was significantly different between HbAS and HbAA children before the start of the *Pf* transmission season (Figure 1a). PBMCs isolated from HbAS children showed a significantly higher production of IFN‐γ than HbAA children (median concentration 1494 pg mL^−1^ *vs.* 362 pg mL^−1^, *P* < 0.0001; Figure 1b). No significant differences were detected between HbAS and HbAA children during the first malaria episode of the ensuing *Pf* transmission season for IFN‐γ or any other cytokine or chemokine (Figures 1c and d).

### The frequency of NK cells is increased in HbAS children before the start of the *Pf* transmission season and correlates with the time to the first malaria episode and with a lower *Pf* density

**Figure 2:**
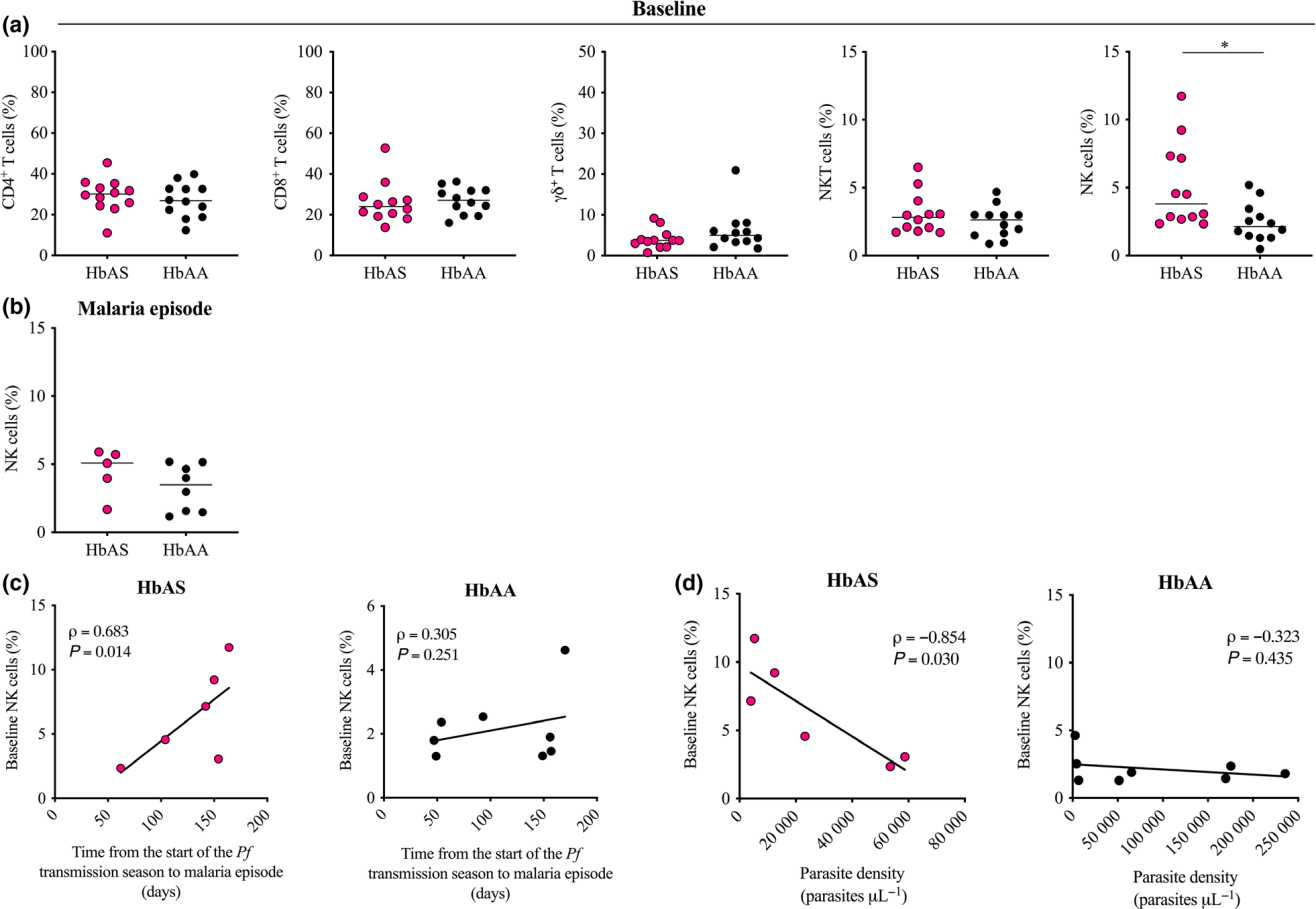
Blood frequency of IFN‐γ‐producing cells in HbAS and HbAA children at baseline and during the first malaria episode of the ensuing *Pf* transmission season. **(a)** Blood frequency of CD4^+^ T cells, CD8^+^ T cells, γδ T cells, NKT and NK cells in HbAS children (*n* = 12) and HbAA children (*n* = 12) at baseline. Normality was assessed by the d’Agostino–Pearson normality test. Groups were compared by either an unpaired *t*‐test or the Mann–Whitney *U*‐test depending on the normality. Median bars are shown. *P < 0.05. **(b)** Blood frequency of NK cells in HbAS children (*n* = 5) and HbAA children (*n* = 8) during the first malaria episode of the ensuing *Pf* transmission season. Normality was assessed by the Shapiro–Wilk normality test. Groups were compared by an unpaired *t*‐test. Median bars are shown. **(c, d)** Correlation between the blood frequency of NK cells at baseline and **(c)** the time from the start of the *Pf* transmission season to the first malaria episode and **(d)** the parasite density quantified during the first malaria episode of the ensuing *Pf* transmission season in HbAS children (**left panel**, *n* = 6) and HbAA children (**right panel**, *n* = 8). Pearson’s correlation coefficients are reported. Technical replicate (*n* = 1).

Since our multiplex screening of cytokine and chemokine production showed that HbAS and HbAA children repeatedly exposed to *Pf* displayed a significantly different IFN‐γ response before the start of the transmission season, we next assessed the frequency of PBMC subsets contributing to the systemic IFN‐γ response. The frequency of CD4^+^ T cells, CD8^+^ T cells, γδ T cells, NKT and NK cells in HbAS and HbAA children was determined by flow cytometry (Supplementary figure 1). There was no significant difference in the frequencies of CD4^+^ T cells, CD8^+^ T cells, γδ T cells or NKT cells between HbAS and HbAA children before the start of the *Pf* transmission season (median frequency 30.1% *vs.* 26.8%, *P* = 0.479 for CD4^+^ T cells; 23.9% *vs.* 27.1%, *P* = 0.362 for CD8^+^ T cells; 3.7% *vs.* 4.9%, *P* = 0.266 for γδ^+^ T cells; 2.8% *vs.* 2.6% *P = *0.292 for NKT cells; Figure 2a). Surprisingly, NK cells were significantly more frequent in HbAS children than in HbAA children (median frequency 3.8% *vs.* 2.1%, respectively, *P* = 0.014, Figure 2a).

We subsequently examined the frequency of NK cells of all available paired samples collected during the first malaria episode of the ensuing transmission season. HbAS and HbAA children displayed similar frequencies of NK cells (median frequency 5.8% *vs.* 3.5%, *P = *0.222; Figure 2b). Because of the unavailability of some HbAS paired samples at the time of the first malaria episode of the ensuing *Pf* transmission season, statistical analysis of the cell dynamic was compromised. Analysis of available samples is presented in Supplementary figure 2a.

Interestingly, the frequency of NK cells before the start of the *Pf* transmission season was significantly correlated with the time from the start of the *Pf* transmission season to the first malaria episode in HbAS children (ρ* = *0.683, *P = *0.014; Figure 2c, left panel), but not in HbAA children (ρ* = *0.305, *P = *0.251; Figure 2c, right panel). Furthermore, the frequency of NK cells before the start of the *Pf* transmission season was significantly inversely correlated with the parasite density quantified during the first malaria episode of the ensuing transmission season in HbAS children (ρ = −0.854, *P* = 0.030; Figure 2d, left panel), but not in HbAA children (ρ = −0.323, *P = *0.435; Figure 2d, right panel). No significant correlation was observed between the frequency of NK cells before the start of the *Pf* transmission season and the axillary temperature determined during the first malaria episode of the ensuing transmission season neither in HbAS children nor in HbAA children (Supplementary figure 3a).

These data demonstrate that HbAS and HbAA children repeatedly exposed to *Pf* differ in their NK cell profile before the start of the *Pf* transmission season and suggest that this distinct cellular profile displayed by HbAS children may predispose them to a relative protection from malaria.

### The frequency of the CD38^high^ subset in NK cells before the start of the *Pf* transmission season correlates with *Pf* density during the first malaria episode of the ensuing transmission season

We have recently reported that the increased expression of CD38, a multifunctional ecto‐enzyme, on CD4^+^ and CD8^+^ T cells is associated with a reduced *Pf* and *Plasmodium vivax* density in controlled human malaria infection studies.^50^, ^51^ Therefore, we investigated whether significant changes could be detected in CD38 expression on NK cells of HbAS children compared to HbAA children. Among CD38^+^ cells, we were able to clearly distinguish two cell populations expressing distinct level of CD38: CD38^dim^ and CD38^high^ (Supplementary figure 1).

**Figure 3:**
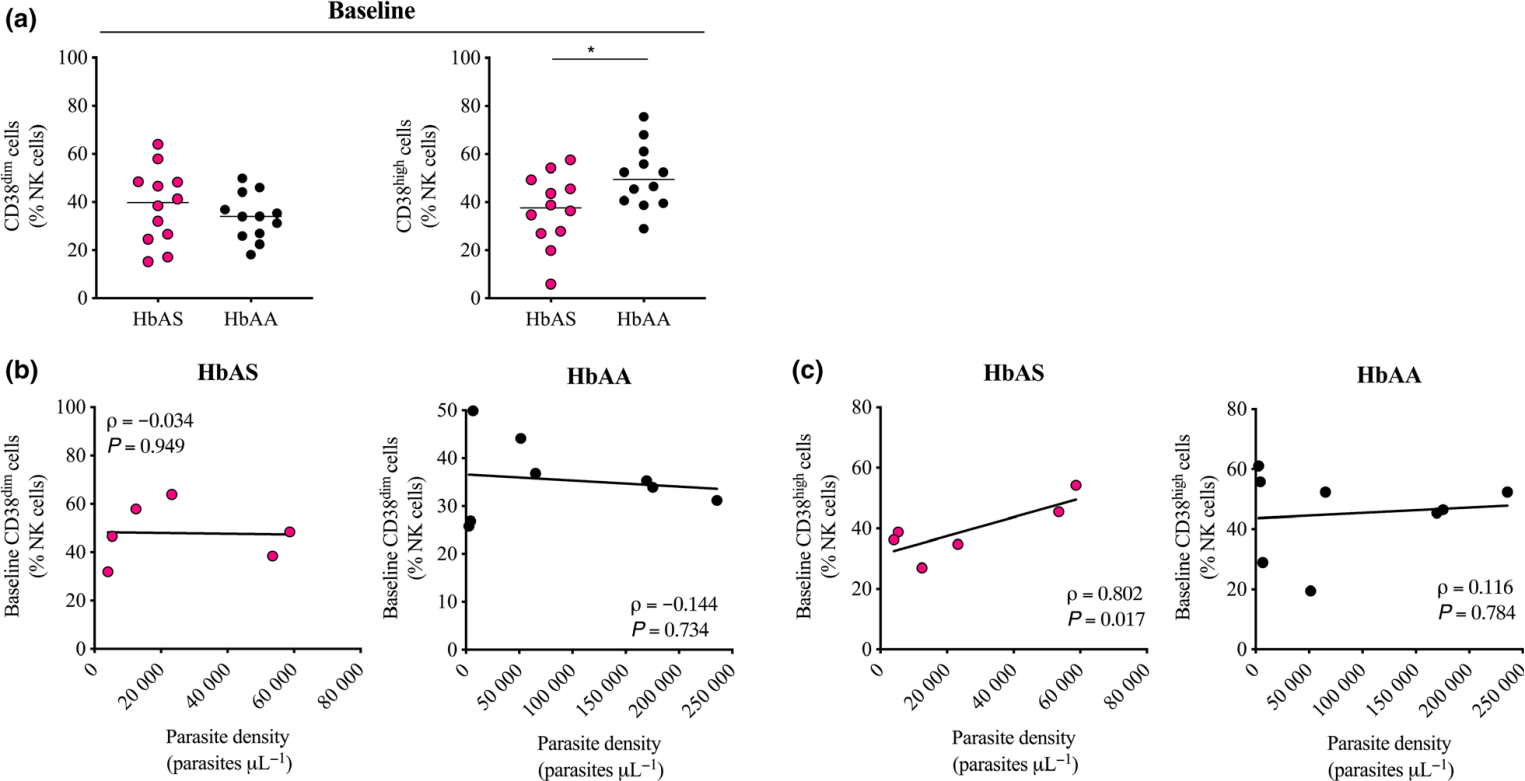
Frequency of CD38^dim^ cells and CD38^high^ cells in NK cells of HbAS and HbAA children at baseline and correlation with the parasite density during the first malaria episode of the ensuing *Pf* transmission season. **(a)** Blood frequency of CD38^dim^ cells **(left panel)** and CD38^high^ **(right panel)** cells in NK cells of HbAS children (*n* = 12) and HbAA children (*n* = 12) at baseline. Normality was assessed by the d’Agostino–Pearson normality test. Groups were compared by an unpaired *t*‐test. Median bars are shown. *P < 0.05. **(b‐c)** Correlation between the blood frequency of either **(b)** CD38^dim^ cells or **(c)** CD38^high^ cells in NK cells at baseline and the parasite density quantified during the first malaria episode of the ensuing *Pf* transmission season in HbAS children (**left panel**, *n* = 6) and HbAA children (**right panel**, *n* = 8). Pearson’s correlation coefficients are reported. Technical replicate (*n* = 1).

We first analysed the contribution of both CD38^dim^ and CD38^high^ subset in the total pool of NK cells. Before the start of the *Pf* transmission season, HbAS and HbAA children did not display significant difference regarding the blood frequency of the CD38^dim^ subset in NK cells (median frequency 39.8% *vs.* 33.9%, *P = *0.389; Figure 3a, left panel), whereas the frequency of the CD38^high^ subset in NK cells of HbAS children was significantly lower in HbAS children (median frequency 37.5% *vs.* 49.4%, *P = *0.027; Figure 3a, right panel). Analysis of the cell dynamic between baseline and the first malaria episode of the ensuing *Pf* transmission season for available samples is presented in Supplementary figure 2b and c.

No significant correlation was observed between the frequency of either the CD38^dim^ or the CD38^high^ subset in NK cells before the start of the *Pf* transmission season and the time from the start of the *Pf* transmission season to the first malaria episode neither in HbAS children nor in HbAA children (Supplementary figure 3b and c). The frequency of the CD38^dim^ subset in NK cells before the start of the *Pf* was not significantly correlated with the parasite density quantified during the first malaria episode neither in HbAS children (ρ = −0.034, *P = *0.949; Figure 3b, left panel) nor in HbAA children (ρ = −0.144, *P = *0.734; Figure 3b, right panel). Also, the frequency of the CD38^high^ subset in NK cells and the parasite density were significantly correlated in HbAS children (ρ* = *0.802 *P = *0.017; Figure 3c, left panel) but not in HbAA children (ρ* = *0.116, *P = *0.784; Figure 3c, right panel), suggesting that the CD38^high^ fraction of NK cells is not involved in the relative protection from malaria observed in HbAS children repeatedly exposed to *Pf*. Similar to our previous observation, none of the two CD38‐expressing subsets in NK cells showed a significant correlation with the axillary temperature neither in HbAS children nor in HbAA children (Supplementary figure 3d and e).

### CD38^dim^ NK cells drive the increased frequency of NK cells in HbAS children before the start of the *Pf* transmission season and are associated with the time to the first malaria episode and with a lower *Pf* density

**Figure 4:**
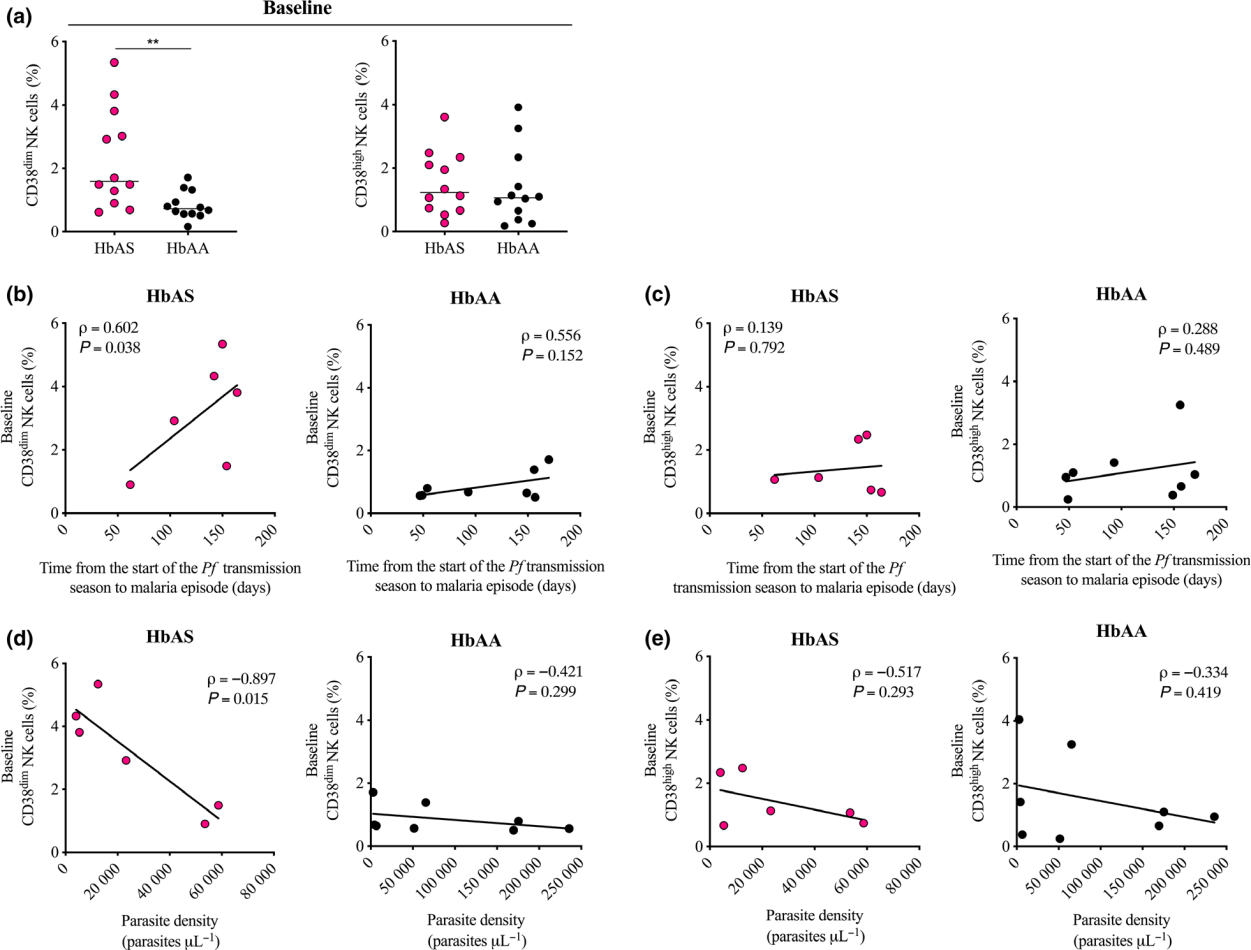
CD38^dim^ and CD38^high^ NK cells of HbAS and HbAA children at baseline, correlation with the time to malaria episode and with the parasite density. **(a)** Blood frequency of CD38^dim^ **(left panel)** and CD38^high^ **(right panel)** NK cells of HbAS children (*n* = 12) and HbAA children (*n* = 12) children at baseline. Normality was assessed by the d’Agostino–Pearson normality test. Groups were compared by an unpaired *t*‐test. Median bars are shown. **P < 0.01. **(b, c)** Correlation between the blood frequency either **(b)** CD38^dim^ NK cells or **(c)** CD38^high^ NK cells at baseline and the time from the start of the *Pf* transmission season to the first malaria episode. **(d, e)** Correlation between the blood frequency either **(d)** CD38^dim^ NK cells or **(e)** CD38^high^ NK cells at baseline and the parasite density quantified during the first malaria episode of the ensuing *Pf* transmission season. **(b–e)** HbAS children (*n* = 6) are shown in the **left panel**, and HbAA (*n* = 8) children are shown in the **right panel**. Pearson’s correlation coefficients are reported. Technical replicate (*n* = 1).

Data presented above show that although the frequency of NK cells as a whole in the circulating blood of HbAS children was increased compared to HbAA children (Figure 2a), the frequency of the CD38^high^ subset in NK cells was significantly lower before the start of the *Pf* transmission season in HbAS children than in HbAA children (Figure 3a, right panel). Thus, the increased frequency of NK cells in the total pool of PBMCs observed in HbAS children was driven by CD38^dim^ NK cells (median frequency 1.6% *vs.* 0.7%, *P = *0.005; Figure 4a, left panel). The frequency of CD38^high^ NK cells was similar in HbAS and HbAA children (median frequency 1.2% *vs.* 1.1%, *P = *0.768; Figure 4a, right panel). Analysis of cell dynamic between baseline and the first malaria episode of the ensuing *Pf* transmission season of available paired samples is provided in Supplementary figure 2d and e.

Remarkably, in HbAS children, we observed a significant correlation between the frequency of CD38^dim^ NK cells before the start of the *Pf* transmission season and the time from the start of the *Pf* transmission season to the first malaria episode (ρ* = *0.602, *P = *0.038; Figure 4b, left panel), but not in HbAA children (ρ* = *0.556, *P = *0.152; Figure 4b, right panel). The frequency of CD38^high^ NK cells was not significantly correlated with the time from the start of the *Pf* transmission season to the first malaria episode neither in HbAS children (ρ = 0.139, *P = *0.792; Figure 4c, left panel) nor in HbAA children (ρ* = *0.288, *P = *0.489; Figure 4c, right panel). Importantly, a significant negative correlation was demonstrated between the frequency of CD38^dim^ NK cells of HbAS children before the start of the *Pf* transmission season and the parasite density quantified during the first malaria episode of the ensuing transmission season (ρ = −0.897, *P = *0.015; Figure 4d, left panel). HbAA children did not display such correlation (ρ = *−*0.421, *P = *0.299; Figure 4d, right panel). Neither HbAS children (ρ = −0.517, *P = *0.293; Figure 4e, left panel) nor HbAA children (ρ = −0.334, *P = *0.419; Figure 4e, right panel) showed a significant correlation between the baseline frequency of CD38^high^ NK cells and the parasite density. None of the two NK subsets showed a significant correlation with the axillary temperature neither in HbAS children nor in HbAA children (Supplementary figure 3f and g).

These data demonstrate that the alteration of the NK cell frequency observed before the start of the *Pf* transmission season in HbAS children repeatedly exposed to *Pf* is driven by CD38^dim^ NK cells and suggest a key role for this cell subset in the sickle‐cell trait‐mediated protection.

### CD38^dim^ NK cells display high IFN‐γ production and reduced cytotoxic potential

**Figure 5:**
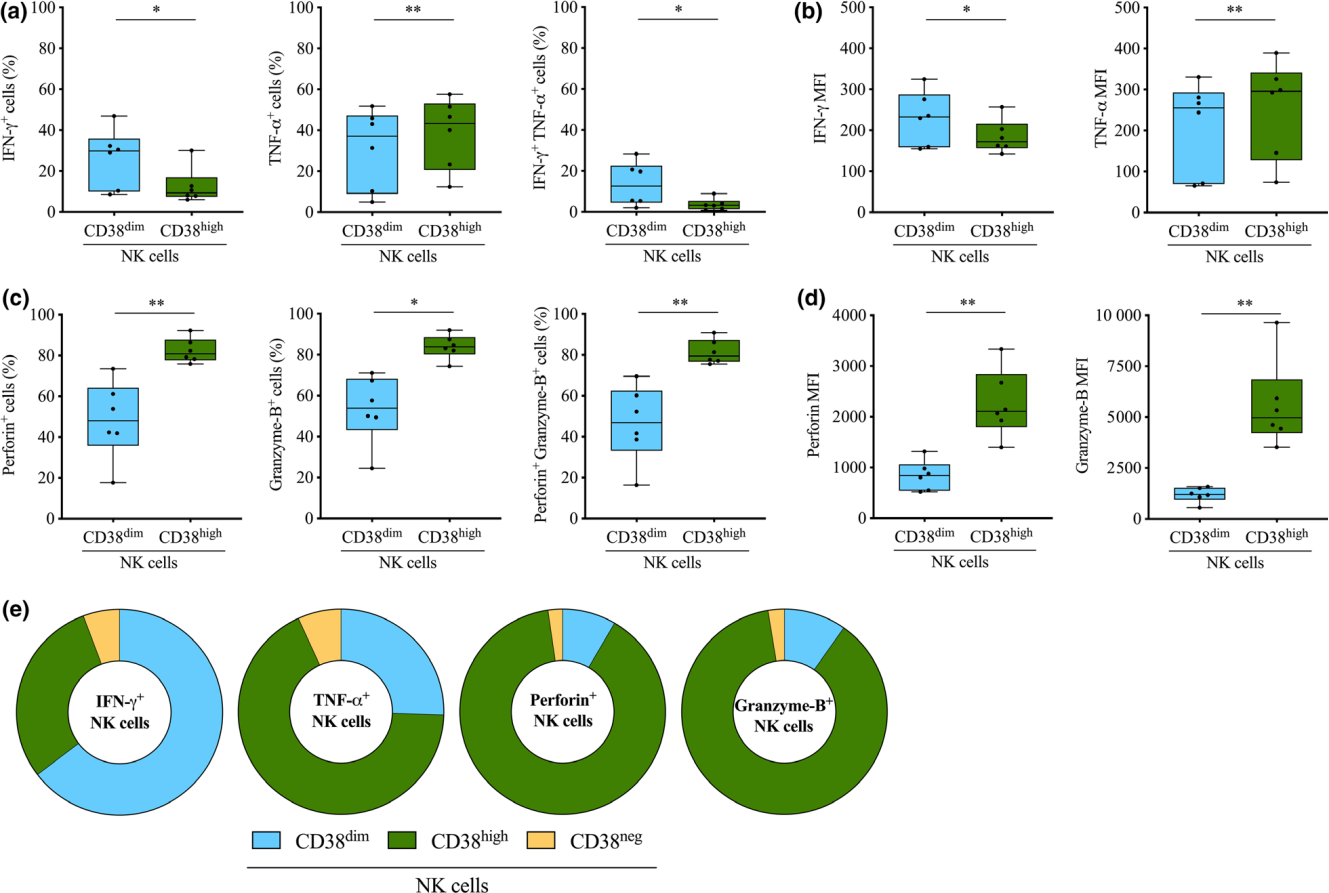
Functional phenotype of CD38^dim^ NK cells and CD38^high^ NK cells. **(a)** Frequency of IFN‐γ^+^ **(left panel)**, TNF‐α^+^ **(middle panel)** and IFN‐γ^+^ TNF‐α^+^ **(right panel)** cells. **(b)** IFN‐γ **(left panel)** and TNF‐α **(right panel)** MFI. **(c)** Frequency of perforin^+^ **(left panel)**, Granzyme‐B^+^ **(middle panel)** and Granzyme‐B^+^ perforin^+^ **(right panel)** cells. **(d)** perforin **(left panel)** and Granzyme‐B **(right panel)** MFI. **(a–d)** Frequency and MFI were determined in CD38^dim^ NK cells (*n* = 6) and CD38^high^ NK cells (*n* = 6). **(e)** Contribution of CD38^dim^ NK cells, CD38^high^ NK cells and CD38^neg^ NK cells in the total pool of IFN‐γ^+^, TNF‐α^+^, perforin^+^ and Granzyme‐B^+^ NK cells (*n* = 6). Normality was assessed by the Shapiro–Wilk normality test. Groups were compared by either an paired *t*‐test or the Wilcoxon’s matched‐pairs signed rank test depending on the normality. *P < 0.05; **P < 0.01. Technical replicate (*n* = 1). MFI, mean fluorescence intensity.

The comprehensive multi‐dimensional flow cytometry analysis of HbAS and HbAA children repeatedly exposed to *Pf* reported above shows that before the start of the *Pf* transmission season, the frequency of the CD38^high^ subset in NK cells was reduced (Figure 3a, right panel), whereas the frequency of CD38^dim^ NK cells in the total pool of PBMCs was increased (Figure 4a, left panel) in HbAS children compared to HbAA. Thus, we next investigated the functional phenotype of the CD38^dim^ and CD38^high^ populations of NK cells. Given the unavailability of additional samples from the HbAS children repeatedly exposed to *Pf* included in this study, we further expanded our investigation of these interesting cell populations to include uninfected malaria‐naïve donors. PBMCs were isolated and then stimulated *in vitro*, and NK cells were segregated for analysis according to their distinct expression of CD38. Measurement of the intracellular expression of IFN‐γ and TNF‐α (Supplementary figure 4) revealed that the CD38^dim^ and CD38^high^ NK cell subsets differed in their cytokine production profiles. Specifically, the frequency of IFN‐γ‐producing cells was significantly higher in the CD38^dim^ NK subset than the CD38^high^ NK subset (median frequency 29.8% *vs.* 9.4%, *P = *0.031; Figure 5a, left panel), whereas the frequency of TNF‐α‐producing cells was significantly reduced in the CD38^dim^ NK subset compared to the CD38^high^ NK subset (median frequency 37.2% *vs.* 43.4%, *P = *0.003; Figure 5a, middle panel). Examination of poly‐functionality revealed that the CD38^dim^ NK cells were significantly enriched in cells co‐expressing IFN‐γ and TNF‐α compared to their CD38^high^ counterparts (median frequency 12.7% *vs.* 3.2%, *P = *0.034; Figure 5a, right panel). Moreover, the production of IFN‐γ as indicated by the mean fluorescence intensity (MFI) was significantly higher in the CD38^dim^ NK subset than the CD38^high^ NK subset (median MFI 233.0 *vs.* 172.5, *P = *0.039; Figure 5b, left panel), whereas the production of TNF‐α was significantly lower in the CD38^dim^ NK subset than the CD38^high^ NK subset (median MFI 255.5 *vs.* 296.0, *P = *0.009; Figure 5b, right panel).

As secretion of cytotoxic granules is also a functional hallmark of NK cells, we next assessed the production of perforin and Granzyme‐B by CD38^dim^ and CD38^high^ NK cells (Figure 5c and d). Consistent with the observed differences between CD38^dim^ and CD38^high^ NK cells with regard to cytokine production, these two subsets also differed in their cytotoxic potential. Specifically, the frequencies of perforin^+^ and Granzyme‐B^+^ cells were significantly lower in the CD38^dim^ NK subset than the CD38^high^ NK subset (median frequency 48.0% *vs.* 80.7%, *P = *0.007 for perforin^+^ cells; and 53.9% *vs.* 83.9%, *P = *0.015 for Granzyme‐B^+^ cells, respectively; Figure 5c, left and middle panel). The CD38^dim^ NK subset contained a significantly lower proportion of cells co‐expressing perforin and Granzyme‐B (46.9% *vs.* 79.5%, *P = *0.005; Figure 5c, right panel). Additionally, the production of perforin in the CD38^dim^ NK subset was approximatively 2.5‐fold lower than in the CD38^high^ NK subset and Granzyme‐B production was lower by approximatively 4.5‐fold (median frequency 841.5 *vs.* 2106, *P = *0.002 for perforin; and 1201 *vs.* 4975, *P = *0.005 for Granzyme‐B, respectively; Figure 5d).

Finally, we determined the contribution of both CD38^dim^ NK subset and CD38^high^ NK subset to the production of IFN‐γ, TNF‐α, perforin and Granzyme‐B in the total pool of NK cells (Figure 5e). The CD38^dim^ NK subset was the major source of IFN‐γ, representing approximatively 65% of the IFN‐γ‐producing NK cell pool. On the contrary, TNF‐α, perforin and Granzyme‐B production in the total pool of NK cells was largely supported by the CD38^high^ NK subset, representing approximatively 68%, 89% and 88% of the productive cells, respectively.

### The CD38^dim^ NK subset is enriched in memory‐activated cells

**Figure 6:**
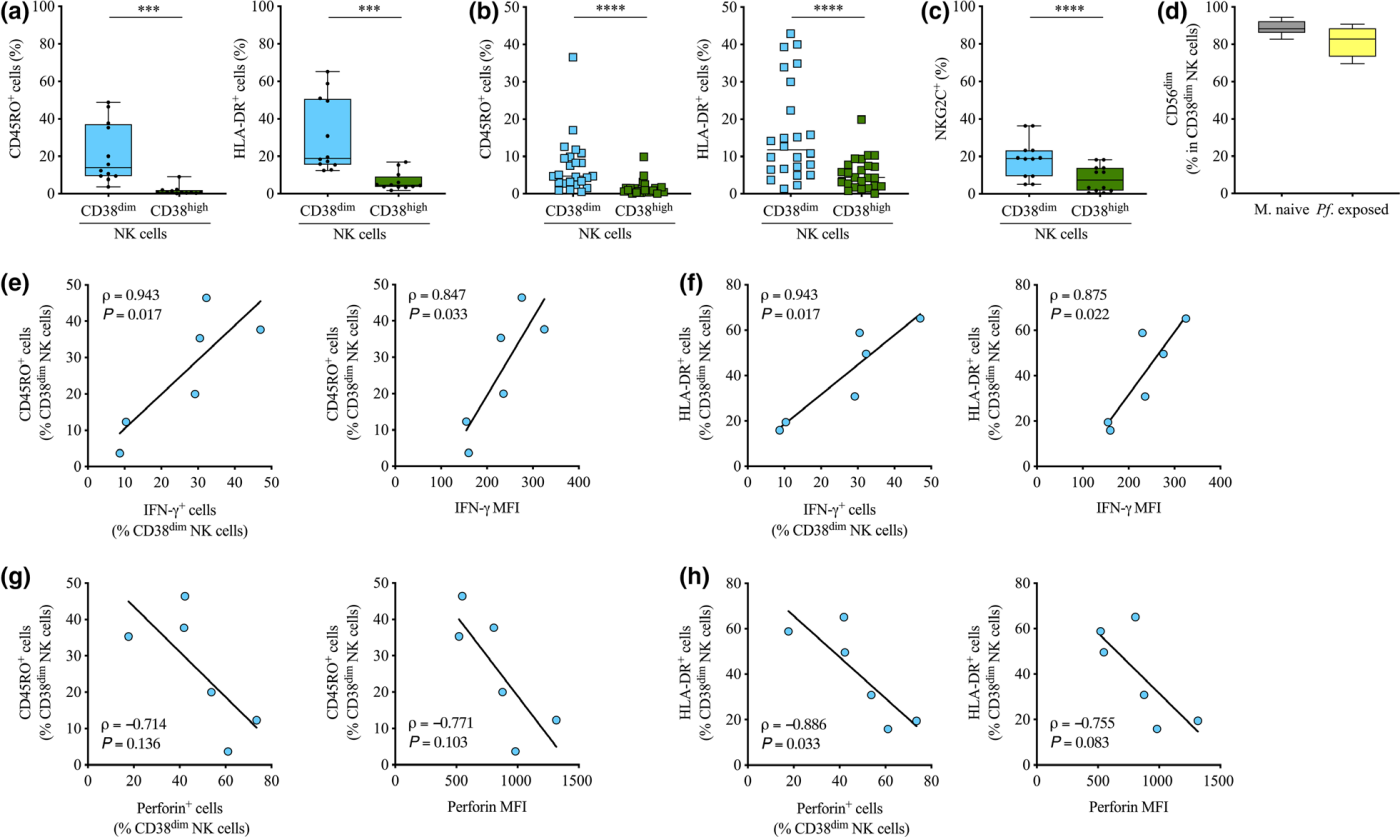
*In vitro* and *in vivo* expression of CD45RO and HLA‐DR on CD38^dim^ NK cells and CD38^high^ NK cells and correlation with production of IFN‐γ and perforin. **(a, b)** *In vitro* **(a)** and *in vivo* **(b)** determination of CD45RO^+^ cell **(left panel)** and HLA‐DR^+^ cell **(right panel)** frequency in CD38^dim^ NK cells (*n* = 12 *in vitro* and *n* = 24 *in vivo*) and CD38^high^ NK cells (*n* = 12 *in vitro* and *n* = 24 *in vivo*). **(c)** Frequency of NKG2C^+^ cells in CD38^dim^ NK cells and CD38^high^ NK cells (*n* = 12). **(d)** Frequency of CD56^dim^ cells in CD38^dim^ NK cells in both malaria‐naïve (*n* = 12) and *Pf*‐exposed individuals (*n* = 24). **(a–d)** Normality was assessed by the d’Agostino–Pearson normality test. Groups were compared by either a paired *t*‐test or the Wilcoxon’s matched‐pairs signed rank test depending on the normality. ***P < 0.001; ****P < 0.0001. **(e, f)** Correlation between the frequency of either **(e)** CD45RO^+^ cells in CD38^dim^ NK cells or **(f)** HLA‐DR^+^ cells in CD38^dim^ NK cells and the frequency of IFN‐γ^+^ cells in CD38^dim^ NK cells **(left panel)**, and IFN‐γ production **(right panel)**, (*n* = 6). Spearman’s correlation coefficients are reported. **(g, h)** Correlation between the frequency of either **(g)** CD45RO^+^ cells in CD38^dim^ NK cells or **(h)** HLA‐DR^+^ cells in CD38^dim^ NK cells and the frequency of perforin^+^ cells in CD38^dim^ NK cells **(left panel)**, and perforin production **(right panel)**, (*n* = 6). Spearman’s correlation coefficients are reported. Technical replicate (*n* = 1).

We next looked in more detail into the phenotype of *in vitro* stimulated malaria‐naïve CD38^dim^ and CD38^high^ NK cells. A strong and rapid production of IFN‐γ and Granzyme‐B following *Mycobacterium* spp. stimulation has been previously linked to the uncommon expression of CD45RO or HLA‐DR on NK cells.^45^, ^52^ Thus, we hypothesised that the high capacity of CD38^dim^ NK cells to generate a strong IFN‐γ response could be associated with expression of memory‐ and/or activation‐associated molecules. Flow cytometry analysis of CD45RO^+^ and HLA‐DR^+^ cells (Supplementary figure 5) revealed significant higher frequency of both cell populations in the CD38^dim^ NK subset than the CD38^high^ NK subset (median frequency 14.0% *vs.* 0.65%, *P = *0.0005 for CD45RO^+^ cells and 19.0% *vs.* 4.6%, *P = *0.0009 for HLA‐DR^+^ cells; Figure 6a). Importantly, these observations were validated *in vivo* in PBMCs collected before the start of the *Pf* transmission season from our cohort of Malian individuals repeatedly exposed to *Pf* (median frequency 4.7% *vs.* 0.8%, *P < *0.0001 for CD45RO^+^ cells and 11.7% *vs.* 4.3%, *P < *0.0001 for HLA‐DR^+^ cells; Figure 6b). CD38^dim^ NK cells were also significantly enriched in cells expressing NKG2C, a NK‐specific activating receptor described on adaptive NK cells in the context of CMV infection,^53^, ^54^, ^55^ as demonstrated by *in vitro* stimulation of malaria‐naïve PBMCs (median frequency 18.9% *vs.* 7.5%, *P* < 0.0001; Figure 6c and Supplementary figure 6). In both uninfected malaria‐naïve donors and *Pf*‐exposed Malian individuals, CD38^dim^ NK cells retained a CD56^dim^ phenotype (median frequency 88.2% in malaria‐naïve individuals and 82.8% in *Pf*‐exposed; Figure 6d).

To further determine to what extent cells expressing memory‐ or activation‐associated molecules were responsible for the cytokine and cytotoxic molecule production detected in the CD38^dim^ NK cell subset, we determined correlation coefficients between the frequency of CD45RO‐ and HLA‐DR‐expressing CD38^dim^ NK cells and the frequency of producing cells, or the production capacity. Importantly, the frequency of the CD45RO^+^ cell subset in CD38^dim^ NK cells was positively correlated with the frequency of IFN‐γ‐producing cells in CD38^dim^ NK cells (ρ* = *0.943, *P = *0.017; Figure 6e, left panel) and the capacity of CD38^dim^ NK cells to produce IFN‐γ (ρ* = *0.847, *P = *0.033; Figure 6e, right panel), suggesting that the CD45RO^+^ subset is the main source of IFN‐γ in CD38^dim^ NK cells. A similar correlation was observed between HLA‐DR^+^ cells in CD38^dim^ NK cells and IFN‐γ‐producing cells in CD38^dim^ NK cells (ρ* = *0.943, *P = *0.017; Figure 6f, left panel) as well as with their IFN‐γ production (ρ* = *0.875, *P = *0.022; Figure 6f, right panel), suggesting that IFN‐γ‐producing CD45RO^+^ HLA‐DR^+^ CD38^dim^ NK cells might constitute a single distinct subset of NK cells. These observations are consistent with the CD45RO^+^ and HLA‐DR^+^ NK subsets being responsible for a robust IFN‐γ response, as noted previously for *Mycobacterium* spp.^45^, ^52^ Remarkably, CD45RO^+^ cells in CD38^dim^ NK cells and HLA‐DR^+^ cells in CD38^dim^ NK cells were both negatively correlated with perforin‐producing CD38^dim^ NK cells (ρ* = −*0.714, *P = *0.136 for CD45RO^+^ cells and ρ* = −*0.886, *P = *0.033 for HLA‐DR^+^ cells; Figure 6g and h, left panel) and perforin production (ρ* = −*0.771, *P = *0.103 for CD45RO^+^ cells and ρ* = −*0.755, *P = *0.083 for HLA‐DR^+^ cells; Figure 6g and h, right panel).

### CD45RO^+^ HLA‐DR^+^ CD38^dim^ NK cells constitute a single distinct subset of NK cells displaying strong IFN‐γ response following stimulation

**Figure 7:**
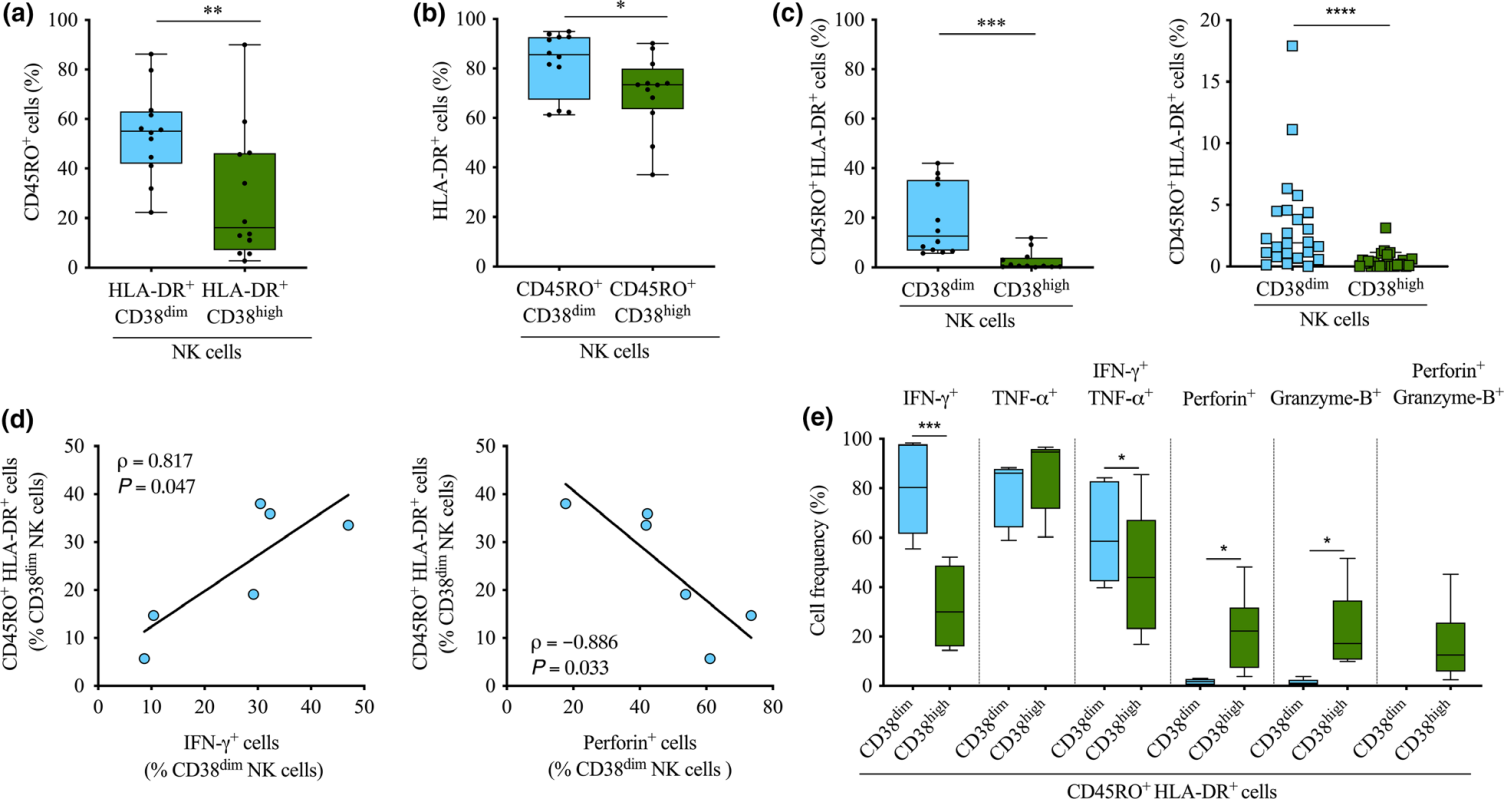
Identification of the CD45RO^+^ HLA‐DR^+^ CD38^dim^ NK cell subset and functional characterisation. **(a)** Frequency of CD45RO^+^ cells in HLA‐DR^+^ CD38^dim^ NK cells (*n* = 12) and HLA‐DR^+^ CD38^high^ NK cells (*n* = 12). **(b)** Frequency of HLA‐DR^+^ cells in CD45RO^+^ CD38^dim^ NK cells (*n* = 12) and CD45RO^+^ CD38^high^ NK cells (*n* = 12). **(c)** Frequency of CD45RO^+^ HLA‐DR^+^ cells in CD38^dim^ NK cells and CD38^high^ NK cells determined *in vitro* (**left panel**, *n* = 12) and *in vivo* (**right panel**, *n* = 24). **(a–c)** Normality was assessed by the d’Agostino–Pearson normality test. Groups were compared by Wilcoxon’s matched‐pairs signed rank test. *P < 0.05; **P < 0.01; ***P < 0.001; ****P < 0.0001. **(d)** Correlation between the frequency of CD45RO^+^ HLA‐DR^+^ cells in CD38^dim^ NK cells and the frequency of IFN‐γ^+^ cells in CD38^dim^ NK cells (**left panel**, *n* = 6) and with the frequency of perforin^+^ cells in CD38^dim^ NK cells (**right panel**, *n* = 6). Spearman’s correlation coefficients are reported. **(e)** Frequency of IFN‐γ^+^ cells, TNF‐α^+^ cells, IFN‐γ^+^ TNF‐α^+^ cells, perforin^+^ cells, Granzyme‐B^+^ cells and Granzyme‐B^+^ perforin^+^ cells in CD45RO^+^ HLA‐DR^+^ CD38^dim^ NK cells and CD45RO^+^ HLA‐DR^+^ CD38^high^ NK cells (*n* = 6). Normality was assessed by the Shapiro–Wilk normality test. Groups were compared by either a paired *t*‐test or Wilcoxon’s matched‐pairs signed rank test depending on the normality. *P < 0.05; ***P < 0.001. Technical replicate (*n* = 1).

We next investigated whether CD45RO^+^ cells and HLA‐DR^+^ cells constituted two distinct subsets of CD38^dim^ NK cells or a single subset co‐expressing both markers. Flow cytometry analysis revealed that HLA‐DR^+^ CD38^dim^ NK cells were significantly enriched in CD45RO^+^ compared to HLA‐DR^+^ CD38^high^ (median frequency 55.2% *vs.* 16.1%, *P* = 0.003; Figure 7a). Similarly, there was a significantly higher frequency of HLA‐DR^+^ cells in CD45RO^+^ CD38^dim^ NK cells than CD45RO^+^ CD38^high^ NK cells (median frequency 85.5% *vs.* 73.4%, *P* = 0.042; Figure 7b). Therefore, the frequency of CD45RO^+^ HLA‐DR^+^ double‐positive cells was evaluated in CD38^dim^ and CD38^high^ NK cells. Our data showed that CD45RO^+^ HLA‐DR^+^ cells were significantly more frequent in CD38^dim^ NK cells than CD38^high^ NK cells (median frequency 12.5% *vs.* 0.6%, *P* = 0.0005; Figure 7c, left panel). Importantly, similar results were also observed *in vivo* in PBMCs collected before the start of the *Pf* transmission season from our cohort of Malian individuals repeatedly exposed to *Pf* (median frequency 1.9% *vs.* 0.2%, *P < *0.0001; Figure 7c, right panel). In accordance with our previous observations, the frequency of CD45RO^+^ HLA‐DR^+^ cells in CD38^dim^ NK cells was significantly correlated with the frequency of IFN‐γ‐producing cells in CD38^dim^ NK cells (ρ* = *0.817, *P = *0.047; Figure 7d, left panel), whereas it was significantly inversely correlated with perforin‐producing CD38^dim^ NK cells (ρ* = −*0.886, *P = *0.033; Figure 7d, right panel).

Finally, we investigated the functional relevance of these correlations. Functional exploration of *in vitro* stimulated malaria‐naïve CD45RO^+^ HLA‐DR^+^ NK cells segregated for the analysis based on CD38 expression (Supplementary figures 4 and 5) revealed a significantly higher frequency of IFN‐γ^+^ cells and IFN‐γ^+^ TNF‐α^+^ cells in CD45RO^+^ HLA‐DR^+^ CD38^dim^ NK cells than their CD38^high^ counterparts (median frequency 80.2% *vs.* 29.9%, *P = *0.0005 for IFN‐γ^+^ cells; 58.6% *vs.* 43.8%, *P = *0.016 for IFN‐γ^+^ TNF‐α^+^ cells; Figure 7e). In contrast, CD45RO^+^ HLA‐DR^+^ CD38^dim^ NK cells displayed a reduced frequency of TNF‐α^+^ cells (median frequency 86.1% *vs.* 94.7%, *P = *0.062; Figure 7e). Consistent with the result reported for the CD38^dim^ NK cell subset as a whole (Figure 5), CD45RO^+^ HLA‐DR^+^ CD38^dim^ NK cells showed a significantly reduced cytotoxic potential as assessed by their frequencies of perforin^+^ cells (median frequency 1.8% *vs.* 22.2%, *P = *0.024; Figure 7e) and Granzyme‐B^+^ cells (median frequency 1.13% *vs.* 17.1%, *P = *0.027; Figure 7e). Additionally, CD45RO^+^ HLA‐DR^+^ CD38^dim^ NK cells contained a lower proportion of cells co‐expressing perforin and Granzyme‐B (median frequency 0.3% *vs.* 12.6%, *P = *0.051; Figure 7e).

Taken together, this study provides the first evidence implicating host cellular immunity in HbAS‐associated immune response to *Pf* infection. We also describe for the first time the existence of a CD38^dim^ NK subset displaying a memory‐activated phenotype and demonstrate that cellular immune profiles enriched with this distinct NK subset before the start of the *Pf* transmission season are associated with better parasite control during a subsequent malaria episode. Our data suggest that these memory‐activated CD38^dim^ NK cells represent an important source of IFN‐γ production, facilitating parasite control in HbAS individuals.

## Discussion

Herein, we report for the first time evidence supporting the existence of a distinct cellular immunity between HbAS and HbAA children repeatedly exposed to *Pf*. Our work points out the existence of a novel NK cell subset that expresses a reduced level of CD38 and co‐expresses high levels of HLA‐DR and CD45RO, representing a memory‐activated phenotype. Functional assessment revealed that the CD38^dim^ CD45RO^+^ HLA‐DR^+^ NK cells represent an important source of IFN‐γ. We further showed that these CD38^dim^ NK cells were specifically enriched in HbAS children compared to HbAA children before the start of the *Pf* transmission season, and were associated with a longer period between the start of the transmission season and the first malaria episode as well as with a better parasite control during this subsequent malaria episode. Therefore, our study suggests that memory‐activated NK cells present before the start of the *Pf* transmission season may contribute to control parasites and delay the onset of the next malaria episode in HbAS children by producing an accelerated and enhanced IFN‐γ response.

There is considerable evidence highlighting the importance of parasite‐induced IFN‐γ responses in the control of *Pf* malaria, including correlation with protection from symptomatic malaria^40^, ^56^ and resistance to *Pf* reinfection.^41^, ^42^ Moreover, the intensity and rapidity of the IFN‐γ response has been associated with both anti‐parasite immunity^57^ and clinical immunity.^58^ Our work demonstrates for the first time that circulating PBMCs isolated before the start of the transmission season from *Pf*‐exposed HbAS individuals display a higher IFN‐γ response to stimulation than HbAA individuals. We further dissected the cellular sources responsible for this early IFN‐γ response. Among all the IFN‐γ‐producing cells explored in our study, we show that NK cells are the only cell subset with a significantly increased frequency in HbAS individuals compared to HbAA before the start of the *Pf* transmission season. More importantly, the frequency of NK cells present before the start of the transmission season in the peripheral blood of HbAS children was significantly correlated with the time from the start of the *Pf* transmission season to malaria episode as well as with the capacity to control parasite density.

Although NK cells are often considered as a bulk population of cells expressing CD56, they constitute a highly heterogeneous cellular compartment of the immune system.^59^, ^60^, ^61^ Similar to T cells, multiple subsets of NK cells can be distinguished based on the expression of a variety of surface receptors and production of immune mediators, with CD56^bright^ NK cells being generally considered as relatively immature cells with high cytokine production capacity, whereas CD56^dim^ NK cells are more mature cells associated with a high cytotoxic activity.^59^, ^62^ Heterogeneity of the NK cell response has been demonstrated in *Pf* infection.^63^ In the present study, by assessing the expression level of the ecto‐enzyme CD38, we identified a CD38^dim^ and a CD38^high^ NK subset. Further analyses demonstrated that CD38^dim^ and CD38^high^ NK cells display a heterogeneous capacity to produce cytokines and cytotoxic mediators. Although in our study both CD38^dim^ and CD38^high^ NK cell subsets were able to produce IFN‐γ, TNF‐α and cytotoxic granules following stimulation, the CD38^dim^ NK cell subset preferentially expressed high levels of IFN‐γ, even though more than 80% of the cells were CD56^dim^. Importantly, the CD38^dim^ NK cell subset was the major source of IFN‐γ in the total pool of NK cells. Such dichotomy between cytokine production and cytotoxic activity has been previously observed on terminally differentiated CD57^+^ NK cells in the context of antiviral response.^64^ Moreover, even if our analysis did not show any significant difference when comparing the frequency of CD56^dim^ cells in CD38^high^ NK cells and CD38^dim^ NK cells of malaria‐naïve individuals (*P* = 0.266; Supplementary figure 7a), we observed that the frequency of CD56^dim^ cells was significantly higher in the CD38^high^ NK subset than CD38^dim^ NK subset of *Pf*‐exposed children (*P* < 0.0001; Supplementary figure 7b). Therefore, the relatively lower proportion of CD56^dim^ cells in the CD38^dim^ NK cell subset could partially explain their reduced capacity to produce cytotoxic granules. Although, we can not exclude that the lower expression of CD38 may also partly explain the reduced release of Perforin and Granzyme‐B by CD38^dim^ NK cells as signalling through this ecto‐enzyme has been previously associated with induction of cytotoxic activity on NK cells.^65^, ^66^

Importantly, we demonstrated that HbAS individuals display a significantly higher frequency of CD38^dim^ NK cells than HbAA individuals before the start of the *Pf* transmission season. Moreover, we show that the enrichment in CD38^dim^ NK cells at baseline is associated with both a delay onset of the next malaria episode and a relative control of parasite density during this episode in HbAS individuals. Previously, a blood‐stage controlled human malaria infection study by our group associated CD4^+^ T cells expressing CD38 with reduced *Pf* density in malaria‐naïve volunteers exposed to *Pf* for the first time^50^, ^51^; however, NK cells were not investigated in that study. Of note, in our current study, analysis of CD4^+^ T cells expressing either CD38^dim^ or CD38^high^ did not reveal any significant difference between HbAS and HbAA children repeatedly exposed to *Pf* (Supplementary figure 8).

The expression of CD45RO at the surface of T cells is conventionally used to discriminate their memory and naïve status, whereas the expression of HLA‐DR is used to assess their activation status. However, CD45RO and HLA‐DR expression on NK cells has been reported.^45^, ^52^, ^67^, ^68^ Herein, we were able to detect both CD45RO and HLA‐DR on the surface of NK cells in both uninfected malaria‐naïve adults and children repeatedly exposed to *Pf*. We further showed that the CD38^dim^ NK subset was particularly enriched in both CD45RO^+^ cells and HLA‐DR^+^ cells as compared to its CD38^high^ counterpart, suggesting that CD38^dim^ NK cells have a memory‐activated phenotype. These exciting findings were supported by the high expression of NKG2C, the CD56^dim^ phenotype and the high cytokine production *versus* reduced cytotoxic potential observed on CD38^dim^ NK cells. Similar to memory mechanisms observed in the T‐cell compartment, establishment and maintenance of the memory‐activated CD38^dim^ NK cell compartment might constitute in HbAS children an adaptive‐like mechanism for optimising the initiation of a very early and strong immune response following *Pf* re‐exposure. In both mice and human, maintenance of memory NK cells has been reported several months post‐infection.^69^, ^70^ To our knowledge, mechanisms supporting the maintenance of memory NK cells in the circulating blood remain unclear. A distinct chemokine receptor profile might allow memory‐activated CD38^dim^ NK cells to patrol through the circulating blood to the peripheral organs.^71^ Also, specific cytokine environment, for example enriched with IL‐15, might support the maintenance of such memory NK cells in the circulating blood.^72^ Alternatively, we cannot exclude that a subclinical infection (below the detection limit of the *Pf*‐PCR^73^) might support the maintenance of a memory‐activated CD38^dim^ NK cell compartment in the blood.

Finally, we identified a CD38^dim^ NK subset co‐expressing both CD45RO and HLA‐DR. Functionally, the expression of either CD45RO or HLA‐DR on NK cells has been previously associated with strong IFN‐γ and cytotoxic responses following stimulation.^45^, ^52^, ^67^ Consistent with those reports, our experiments showed that CD45RO^+^ HLA‐DR^+^ CD38^dim^ NK cells exerted high IFN‐γ responses following stimulation. However, in contrast to previous studies on CD45RO^+^ NK cells and HLA‐DR^+^ NK cells, we observed a reduced cytotoxic response on CD45RO^+^ HLA‐DR^+^ CD38^dim^ NK cells, possibly because of their reduced expression of CD38^65^, ^66^ and/or their more mature status.^64^

The existence of memory NK cells was first established in rodent models.^74^, ^75^, ^76^ The importance of such NK cells has since been highlighted in non‐human primates infected with the simian–human immunodeficiency virus^77^ and in human infected by *Mycobacterium tuberculosis* ^45^ or CMV^53^, ^54^, ^55^; as well as in vaccinated individuals.^46^, ^52^, ^78^ Recently, a high frequency of NK^dim^ NKG2C^+^ CD57^+^ memory‐like cells at the time of primary HIV infection was associated with an enhanced early control of the viral load under combined antiretroviral therapy.^79^ In the context of *Pf* infection, the expression of the DNAX accessory molecule 1, a co‐stimulatory molecule found essential for the differentiation of memory NK cells,^80^ was associated with the NK ability to eliminate parasitised red blood cells.^81^ More recently, the frequency of an antibody‐dependent memory‐like NK cell subset has been correlated with low parasitaemia and resistance to malaria.^49^

Herein, by identifying the existence of a novel memory‐activated NK cell subset characterised by the CD38^dim^ CD45RO^+^ HLA‐DR^+^ phenotype, we show for the first time the involvement of cellular immunity in the HbAS‐associated immune response to *Pf* infection. Our results show that the enrichment in CD38^dim^ NK cells before the start of the *Pf* transmission season plays a key role in the better parasite control observed in HbAS children during the first malaria episode of the ensuing transmission season. Our study has important implications for the understanding of immunity to malaria and the development of effective therapeutic interventions. These data suggest that the induction of an adaptive/memory NK response should be considered in the design of new vaccine candidates against *Plasmodium* infection.

## Methods

### Subjects

Subjects included in the study presented herein were a subset from individuals aged 3 months to 25 years, enrolled in a previously described cohort study.^82^ Briefly, a prospective cohort study was conducted in Kalifabougou, Mali, where intense seasonal *Pf* transmission occurs during a predictable 6‐month period from July through December and where repeated infections are common.^82^ At baseline (before the start of the *Pf* transmission season), all subjects displayed a haemoglobin level > 7 g dL^−1^, an axillary temperature ≤ 37.5°C and were free of acute systemic illness and chronic disease. Malaria episodes were detected by passive (self‐reporting to local health centre) and active (medical examination and PCR, every 2 weeks from enrolment through December) surveillance. Malaria episodes were defined as parasitaemia ≥ 2500 parasites μL^−1^, an axillary temperature > 37.5°C and/or signs and symptoms consistent with malaria.^82^

Here, we focus on individuals aged 7–13 years, which is the age range during which the acquisition of clinical immunity to uncomplicated malaria begins in areas of intense malaria transmission. From this subset, we randomly selected 12 HbAA and 12 HbAS children free of *Pf* infection (as by PCR), with blood samples and clinical data available before the start of the *Pf* transmission season (Table 1). At the first malaria episode of the ensuing transmission season, paired clinical data were available for 8 HbAA and 6 HbAS individuals (Table 1) and paired blood samples were available for 8 HbAA and 5 HbAS individuals. The four groups were similar in terms of gender ratio (*P* = 0.551), body weight (*P* = 0.460), age (*P* = 0.741) and haemoglobin concentration (*P* = 0.225) (Table 1). During the first malaria episode, the axillary temperature was significantly increased in HbAA children (*P* = 0.003; Table 1) and HbAS children (*P* = 0.025; Table 1) compared to baseline.

**Table 1:** Clinical characteristics

	Baseline		Malaria episode		*P* value
	HbAA	HbAS	HbAA	HbAS	
	(*n* = 12)	(*n* = 12)	(*n* = 8)	(*n* = 6)	
Gender ratio (F/M)	1.00	0.50	1.67	0.50	NS^b^
Body weight (kg)^a^	20.0 [17–40]	23.0 [19–34]	21.5 [17–29]	23.0 [19–37]	NS^c^
Axillary temperature (°C)^a^	36.4 [35.9–36.8]	36.5 [36.0–37.1]	38.4 [36.0–39.8]	37.3 [37.0–39.0]	HbAA* P* < 0.01^d^ HbAS *P* < 0.05^d^
Age (years)^a^	8 [7–12]	9 [7–13]	8.5 [7–11]	8.5 [7–13]	NS^c^
Haemoglobin concentration (g dL^−1^)^a^	12.3 [10.1–13.9]	11.5 [10.6–13.9]	11.6 [9.6–14.6]	11.9 [10.0–13.4]	NS^e^
Parasite density (parasites μL^−1^)^a^			58 425 [2725–235 725]	17 850 [4050–58 700]	NS^f^

*P* < 0.05 indicates a significant difference in axillary temperature measured at baseline and during malaria episode in HbAA individuals.

F, female; Hb, haemoglobin; M, male; NS, not significant.

^a^ Median values are reported; range is indicated in square brackets.

^b^ Chi‐square test.

^c^ Kruskal–Wallis test.

^d^ Paired *t*‐test.

^e^ one‐way ANOVA.

^f^ Unpaired *t*‐test with Welch’s correction.

### Samples

Venous blood (≤ 8 mL) was collected at baseline and at the time of the first malaria episode into sodium heparin tubes (BD Vacutainer CPT, Franklin Lakes, NJ) and transported to the local laboratory for processing within 3 h of collection. PBMCs were isolated by standard density gradient centrifugation and stored at −80°C overnight before long‐term storage in liquid nitrogen.

### Haemoglobin type

Haemoglobin typing for HbA, HbS and HbC was determined by high‐performance liquid chromatography using a D‐10 haemoglobin analyser (Bio‐Rad, Hercules, CA).

### Parasite density

Parasite density was quantified from genomic DNA of *Pf* asexual parasites extracted from dried blood spots as previously described.^73^ Briefly, the *Pf* 18S rRNA gene was amplified by 15‐cycle standard PCR amplification followed by a qPCR. The limit of detection of the nested qPCR was ~0.5 parasites μL^−1^.

### Cytokine and chemokine quantification

Concentrations of IFN‐γ, IL‐1β, IL‐6, IL‐12p70, IL‐17A, IP‐10, MCP‐1, MIP‐1α, MIP‐1β and TNF‐α were quantified in the supernatant of PBMCs stimulated with 20 ng mL^−1^ of PMA and 1000 ng mL^−1^ of Ionomycin (Sigma‐Aldrich, St. Louis, MO) for 6 h at 37°C by using the Human ProcartaPlex Panel (Invitrogen, Carlsbad, CA) following the manufacturer’s instructions. Detection was performed using a MagPIX analyser driven by xPONENT software (Luminex Corporation, Austin, TX).

### Immunophenotyping of peripheral blood lymphocytes

Peripheral blood mononuclear cells isolated from peripheral blood samples from HbAS and HbAA children were stimulated with 20 ng mL^−1^ of PMA and 1000 ng mL^−1^ of Ionomycin (Sigma‐Aldrich) for 6 h at 37°C. Cells were then stained for 20 min with phenotype‐associated markers with anti‐human CD3‐APC‐H7 (SK7), CD4‐BUV495 (SK3), CD56‐PE (B159) (all from BD Biosciences, Franklin Lakes, NJ), CD8‐Pe‐Cy7 (SK1; BioLegend, San Diego, CA) and TCRγδ‐FITC (5a6E9; Invitrogen); and for activation/memory‐associated markers with anti‐human CD45RO‐BV510 (UCHL1; BioLegend) and HLA‐DR‐PerCP‐Cy5.5 (G46‐6; BD Biosciences); and with anti‐human CD38‐BV650 (HB‐7; BioLegend), monoclonal antibodies (mAbs). Samples were then analysed using a BD LSRFortessa X20 driven by FACSDiva software (BD Biosciences). FlowJo software version 10.4 was used for gating. Because of the limited availability of samples for some subjects at their first malaria episode, flow cytometry analyses could not be performed on the entire study population.

### *In vitro* functional profile of NK cells

Peripheral blood mononuclear cells isolated from malaria‐naïve buffy coats were stimulated with 20 ng mL^−1^ of PMA and 1000 ng mL^−1^ of Ionomycin (Sigma‐Aldrich) for 1 h at 37°C. Monensin A (GolgiStop; BD Biosciences) was added, and cells were incubated for a further 5 h at 37°C. Stimulated cells were then stained with anti‐human CD3, CD56, HLA‐DR, CD45RO and CD38 mAbs (as described above) for 20 mins; fixed and permeabilised using the Cytofix/Cytoperm kit (BD Biosciences) following the manufacturer’s instructions; and then stained with either anti‐human IFN‐γ‐FITC (4S.B3; BD Biosciences) and TNF‐α‐BV785 (Mab11; BioLegend), or perforin‐FITC (B‐D48) and Granzyme‐B‐AF647 (QA16A02) (both BioLegend) mAbs. Functional profile of NK cells was analysed on a BD LSRFortessa X20 driven by FACSDiva software (BD Biosciences). FlowJo software version 10.4 was used for gating.

### NKG2C profile of NK cells

Peripheral blood mononuclear cells isolated from malaria‐naïve buffy coats were stimulated with 20 ng mL^−1^ of PMA and 1000 ng mL^−1^ of Ionomycin (Sigma‐Aldrich) for 6 h. Stimulated PBMCs were stained with CD3, CD56, HLA‐DR, CD45RO, CD38 (as described above) and NKG2C‐VioBright FITC (REA205; Miltenyi Biotec, Bergisch Gladbach, Germany) mAbs for 20 min. Cells were analysed as described above.

### Statistical analysis

Statistical analyses were performed with Prism 7 (GraphPad Software, Inc., San Diego, CA). Statistical tests used are indicated for each Table and Figure. Normality was assessed by either the d’Agostino–Pearson normality test or the Shapiro–Wilk normality test, if ‘*n*’ is too small. *P*‐values < 0.05 were considered statistically significant.

### Study approval

The Malian cohort study was conducted in compliance with all applicable federal regulations governing protection of human subjects and was approved by the Ethics Committee of the Faculty of Medicine, Pharmacy and Dentistry at the University of Sciences, Technique and Technology of Bamako and the Institutional Review Board of the National Institute of Allergy and Infectious Diseases, National Institutes of Health. The study is registered on http://www.clinicaltrials.gov (NCT01322581). Written informed consent was obtained from the parents or guardians of participating children. The laboratory study with samples collected during that study was approved by the James Cook University Human Research Ethics Committee (#H7735). Subjects with no reported history of malaria or malaria exposure (malaria‐naïve, *n* = 6) were provided by the Australian Red Cross under a protocol approved by the James Cook University Human Research Ethics Committee (#H6702); written informed consent was obtained from all subjects.

## Conflict of interest

The authors have declared that no conflict of interest exists.

## Author contributions

CL, CP and DLD contributed to the project design. CL processed the samples, performed the experiments and analysed the data. OKD, BT and PDC recruited participants and provided samples. JLB organised import permits and assisted with the laboratory studies. KPDS, DLD and BT coordinated the shipment of samples. CL and DLD wrote the manuscript.

## Supporting information

Supplementary figure 1

Supplementary figure 2

Supplementary figure 3

Supplementary figure 4

Supplementary figure 5

Supplementary figure 6

Supplementary figure 7

Supplementary figure 8
