# Supplementary figures and images for: A novel population of memory‐activated natural killer cells associated with low parasitaemia in Plasmodium falciparum‐exposed sickle‐cell trait children
Source: Clin Transl Immunology. 2020 Apr 2;9(4):e1125. doi: 10.1002/cti2.1125 (PMC7114700; doi:10.1002/cti2.1125)

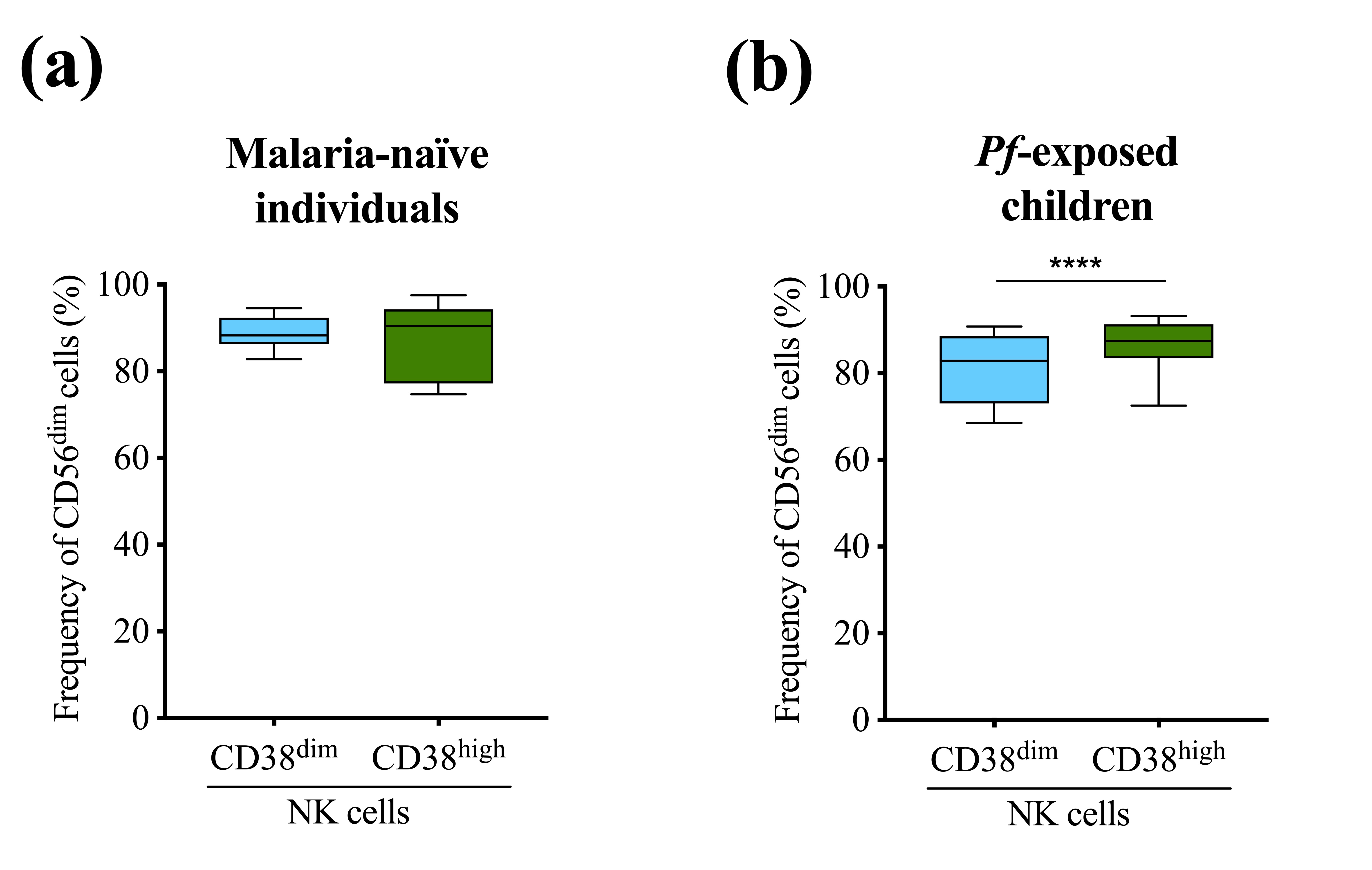

Supplement: Supplementary file 7 — Supplementary figure 7 [file CTI2-9-e1125-s007.jpg]

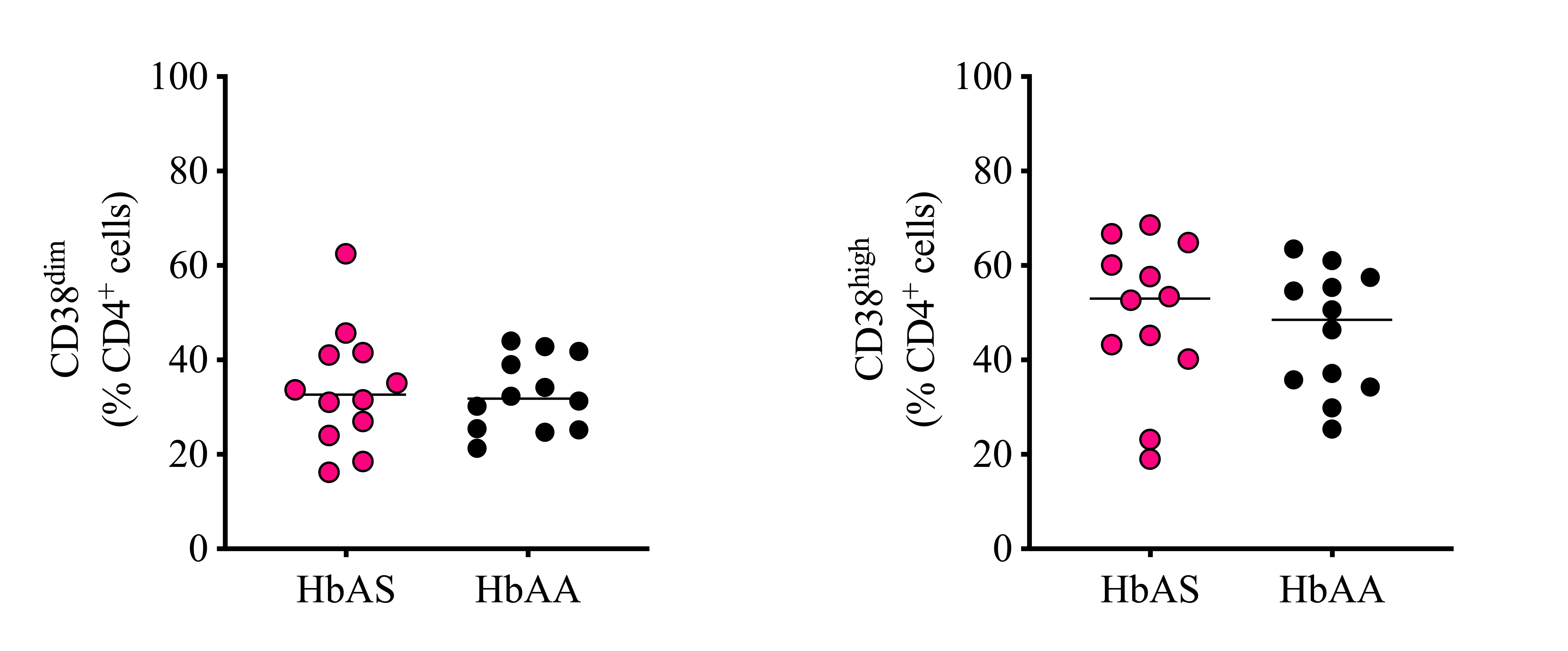

Supplement: Supplementary file 8 — Supplementary figure 8 [file CTI2-9-e1125-s008.jpg]
